# Small Cell and Large Cell Neuroendocrine Carcinoma of the Colon and Rectum: Population-Based Analysis of Incidence, Survival, and Site-Specific Outcomes

**DOI:** 10.3390/cancers18121976

**Published:** 2026-06-18

**Authors:** Nora Y. Sun, Alexander H. Xiao, Thorvardur R. Halfdanarson, Timothy J. Hobday, Patrick W. McGarrah, Conor D. J. O’Donnell, Qian Shi, Mohamad B. Sonbol, Nguyen H. Tran, Zhaohui Jin

**Affiliations:** 1 Department of Statistics, Harvard University, Cambridge, MA 02138, USA; norasun@college.harvard.edu; 2 Division of Oncology, Mayo Clinic, Rochester, MN 55905, USA; 3 Division of Oncology, Mayo Clinic, Jacksonville, FL 32224, USA; 4 Division of Oncology, Mayo Clinic, Scottsdale, AZ 85259, USA

**Keywords:** colorectal small cell neuroendocrine carcinoma, colorectal large cell neuroendocrine carcinoma

## Abstract

Background: Colorectal neuroendocrine carcinomas (NECs), including small cell (SCNEC) and large cell (LCNEC) subtypes, are rare but aggressive malignancies with limited population-level data. Moreover, prior studies have limited stratification by histologic subtype and anatomic location, despite evidence of disease heterogeneity in colorectal malignancies. This study aimed to characterize the clinical characteristics, treatment patterns, and outcomes of colorectal NEC by histologic subtype and tumor location, and to compare them with neuroendocrine tumors (NETs) and adenocarcinomas (ACs). Methods: A retrospective cohort review was conducted utilizing the SEER database from 2000 to 2022 to identify patients with colorectal SCNEC, LCNEC, NET, and AC. Demographic, clinical, therapeutic and survival data were analyzed and compared using Kaplan–Meier methods, log-rank tests, and Cox proportional hazards regression. Results: A total of 790 SCNEC, 498 LCNEC, 31,200 NET, and 638,898 AC cases were identified. SCNEC and LCNEC were associated with advanced stage at diagnosis (66% and 55% stage IV, respectively) and inferior survival outcomes compared to NET and AC (median OS (mOS) of 7 and 8 months for SCNEC and LCNEC, respectively, *p* = 0.0002). Stratified by location, colonic SCNEC had the poorest survival (mOS 5 months), worse than rectal SCNEC (mOS 9 months); this pattern was not observed in LCNEC. Surgical resection was associated with improved OS in both NEC subtypes (HR range 0.25–0.56, all *p* < 0.012). Radiation therapy was associated with improved survival in NEC overall (SCNEC HR 0.58; LCNEC HR 0.71, both *p* < 0.05), with the observed benefit appearing greatest in rectal NEC. Demographic factors were associated with survival in NET and AC but had no significant impact in NEC. Conclusions: Colorectal SCNEC and LCNEC are highly aggressive malignancies with poor outcomes, with SCNEC demonstrating modestly inferior outcomes compared to LCNEC. This study demonstrates that both histologic subtype and anatomic site of disease are important determinants of prognosis and treatment response. This supports the concept that colorectal NEC represents a biologically heterogeneous disease, with colonic SCNEC showing the worst outcomes and rectal NEC associated with a potential enhanced response to radiation. These findings support the need for further prospective and molecular studies to better define treatment approaches and targeted therapies for these rare malignancies.

## Simple Summary

Colorectal neuroendocrine cancers (NECs), including small cell (SCNEC) and large cell (LCNEC) subtypes, are rare but aggressive cancers with limited data regarding their clinical behavior and treatment outcomes. A large national database was used to evaluate patients with SCNEC and LCNEC, comparing outcomes both by subtype and location in the colorectal tract. NEC patients presented with more advanced disease and had worse outcomes than more common colorectal cancers. Patients with SCNEC demonstrated slightly poorer survival than those with LCNEC, particularly in the colon. Surgery was associated with longer survival in both NEC subtypes, while radiation was modestly associated with improved outcomes in rectal NEC. These findings highlight the important differences between NEC by subtype and tumor location and support the need for further research to guide more tailored treatment strategies.

## 1. Introduction

Neuroendocrine carcinomas are a group of rare and highly aggressive neoplasms, characterized by their unique morphology and elevated markers of proliferation, with high mitotic rates or Ki-67 proliferation index [1]. Small cell neuroendocrine carcinoma (SCNEC) and large cell neuroendocrine carcinoma (LCNEC) are two poorly differentiated subtypes within neuroendocrine carcinomas [2]. SCNEC and LCNEC occur primarily in the lungs and account for 15–20% and 3% of lung cancers, respectively; less than 5% of SCNECs and LCNECs are extrapulmonary [2,3]. While colorectal NEC is a common extrapulmonary NEC, it comprises less than 1% of colorectal cancers, although several recent studies have found that incidences of colorectal NEC are rising [4,5,6]. Outcomes of colorectal NEC are poor, with a median overall survival (mOS) of less than a year, with 5-year survival rates of 22% for colorectal LCNEC and 9% for colorectal SCNEC [5,7].

Several recent studies have found differences in molecular biology between colorectal SCNEC and LCNEC; however, the clinical impact of these molecular differences between these subtypes remains largely unstudied [8,9,10]. There appear to be genetic similarities between colorectal NEC and colorectal adenocarcinoma, with mutations in APC, KRAS, BRAF, and FBXW7 commonly observed in both [8,11,12]. Mutations in BRAF and KRAS have been associated with worse outcomes but to date, no data exist yet to support the use of targeted therapy in patients with BRAF V600E mutated colorectal NECs [13,14]. The management of colorectal NEC is extrapolated from existing small cell carcinoma of the lung or colorectal adenocarcinoma (CRC AC) protocols, with most treatment assessments limited to case reports and single institution retrospective studies [15,16]. Given the genetic differences between colorectal NEC and SCLC, it is not surprising that the outcomes of SCLC-like therapy have yielded disappointing results and in recent years, the use of CRC AC protocols has increased, but supportive data are still extremely limited.

Given the rarity of colorectal NEC, most prior studies have not stratified by SCNEC versus LCNEC nor by the site of the primary tumor. Thus, important differences in clinical behavior and treatment outcomes in these subtypes may remain unrecognized. Therefore, the primary outcome of this study was to characterize the demographic features, clinical characteristics, treatment patterns, and outcomes of colorectal NEC utilizing a large population-based cohort. Secondary objectives were to compare outcomes between SCNEC and LCNEC, evaluate differences between colonic and rectal primaries, and compare NEC outcomes with colorectal adenocarcinomas (ACs) and neuroendocrine tumors (NETs).

## 2. Methods

The Surveillance, Epidemiology, and End Results (SEER) database “Incidence—SEER Research Plus Data, 17 Registries, Nov 2024 Sub (2000–2022), Malignant Behavior, Known Age” was utilized to identify patients from 2000 to 2022 ICD-O-3 using histologic codes corresponding to SCNEC, LCNEC, NET, or adenocarcinoma and “Site recode ICD-O-3/WHO 2008” of appendix, cecum, ascending colon, hepatic flexure, transverse colon, splenic flexure, descending colon, sigmoid colon, rectosigmoid junction, and rectum. The SEER database is a population-based cancer registry administered by the United States National Cancer Institute that collects demographic, incidence, treatment, and survival data from multiple geographic regions across the United States. Data were extracted using SEER*Stat 9.0.41 in August 2025. Patients with missing survival data or essential staging information were excluded. AJCC stage was calculated with 8th ed. TNM classification. Vital status (alive/dead) was determined in SEER by the date of last follow-up. The primary outcome measured was overall survival (OS).

A series of statistical analyses were performed within each of the four histologic groups. *T*-tests, Chi-squared tests, and Fisher’s exact tests were used to compare demographic and clinical characteristics associated with colon and rectal primaries. Univariate and multivariate Cox regressions were performed with demographic and clinical variables including age at diagnosis, gender, ethnicity, median household income, year of diagnosis, stage at diagnosis, surgical codes for primary surgery site, receipt of radiation therapy, and site of disease. Multivariate Cox regressions only included variables that were significant in a univariate Cox regression. Given that the AJCC stage incorporates TNM staging, Cox regression models for AJCC and TNM staging were run separately.

To compare survival comparisons between the four histologic groups, Kaplan–Meier and log-rank tests were performed. These non-parametric methods appropriately account for censored data without requiring assumptions regarding the underlying survival distribution, and provide visual representation of hazard ratios between histologic subtypes over time. Cases lost to follow-up were censored. All statistical analyses were performed using R 4.4.2 in the 2025 version of RStudio.

## 3. Results

### 3.1. Patient Demographics and Disease Characteristics

Within SEER patients with colorectal tumors, 790 patients with SCNEC, 498 with LCNEC, 31,200 with NET, and 638,898 with AC from 2000 to 2022 were identified. Male patients represented 55% of SCNECs, 51% of LCNECs, 47% of NETs, and 53% of ACs. Most patients were White (84% SCNEC, 83% LCNEC, 67% NET, and 80% AC).

The median age in years at diagnosis was 65 for SCNEC, 68 for LCNEC, 53 for NET, and 67 for AC. At diagnosis, 520 (66%) of SCNEC and 274 (55%) of LCNEC patients were stage IV, compared with 553 (2%) of NET and 114,110 (18%) of AC patients. While 303,488 (55%) of AC patients presented with stage I or II disease, only 80 (10%) of SCNEC patients did so. Surgery was performed for 287 (36%) SCNEC patients compared to 368 (74%) of LCNEC patients. Radiation was administered to 188 (24%) and 53 (11%) SCNEC and LCNEC patients, respectively (Table 1).

### 3.2. Disease Outcomes

Median OS accounting for all stages was 7 months for SCNEC, 8 months for LCNEC (*p* = 0.0002), and was not achieved for NETs or AC. Within stage IV disease, mOS was 5 months for SCNEC and LCNEC, 46 months for NETs, and 16 months for AC. Across all subtypes, higher AJCC stage and not undergoing surgical resection were associated with reduced OS. Among patients with SCNEC and LCNEC, there was no impact on OS with regard to any demographic feature including age, gender, ethnicity or household income (Table 2). Initiation of systemic therapy was associated with longer mOS for both SCNEC (18 vs. 6 months, *p* < 0.0001) and LCNEC (16 vs. 5 months *p* < 0.0001).

### 3.3. Effect of Disease Location

Between colonic and rectal primaries, SCNEC was distributed evenly at 48% and 52%, respectively. In contrast, 81% of the LCNEC cases presented with colonic disease. Taking all stages into account, patients with rectal SCNEC had a mOS of 9 months, compared to 5 months in colonic SCNEC (*p* < 0.0001). In contrast, in the LCNEC cohort there was no survival difference between rectal and colonic primary. When matching stage and site of disease between LCNEC and SCNEC, only the pair of stage IV colonic SCNEC and LCNEC had a statistically significant difference in mOS, at 2 and 5 months respectively (*p* < 0.0001, Table 3).

Patients with rectal SCNEC were younger than those with colonic SCNEC (mean 62 vs. 66 years, *p* = 0.0004) and had fewer distant metastases (61% vs. 72%, *p* = 0.003). Accounting for all stages, patients with stage IV rectal SCNEC had an improved mOS of 7 months compared to 2 months in stage IV colonic SCNEC (*p* < 0.0001). Patients with rectal SCNEC received surgical intervention less frequently than colonic SCNEC (19% vs. 57%, *p* < 0.0001) but received radiation more frequently (38% vs. 6%, respectively, *p* < 0.0001).

Patients with rectal LCNEC were also younger than patients with colonic LCNEC (mean 64 vs. 67 years; *p* = 0.03) and were more often male (60% vs. 48%, *p* = 0.03). Compared to rectal LCNEC, patients with colonic LCNEC more frequently presented with stage IV disease (58% vs. 53% *p* = 0.05), T4 disease (35% vs. 15% *p* = 0.0005), and N2 disease (41% vs. 19% *p* = 0.0005). There were no statistically significant differences in mOS for LCNEC when stratified by site of disease. Patients with rectal LCNEC underwent surgical resection less frequently than patients with colonic LCNEC (47% vs. 82%, *p* < 0.0001), but received radiation therapy more frequently (34% vs. 3%; *p* < 0.0001).

Undergoing surgery was associated with a statistically significant increased OS for colonic and rectal SCNECs, LCNECs, ACs, and NETs (among NEC, HR range 0.25–0.56, all *p* < 0.012). Receiving radiation was associated with an increased OS for both SCNEC and LCNEC, with HRs of 0.58 and 0.71, respectively, (*p* < 0.05). When stratified by site, with multivariate analysis, there was no impact of radiation on OS for colonic SCNEC or LCNEC. However, among patients with rectal SCNEC and LCNEC, receipt of radiation was associated with a lower risk of mortality (HR = 0.79, *p* = 0.06 and HR = 0.59, *p* = 0.07 respectively, Table 4).

### 3.4. Colorectal Adenocarcinomas and Neuroendocrine Tumors

Surgery was performed for 85% of both NET and AC patients, more frequently than for either NEC (36% of SCNEC and 74% of LCNEC patients). Radiation was administered to 152 (0.5%) of NET and 94,097 (15%) of AC patients (Supplemental Table S1). In patients with AC and NET, female sex, “other” ethnicity, and increased household income were associated with increased OS; these demographic variables had no impact on OS in NEC. In patients with ACs, those older than 55 had reduced OS compared to those <40 years old (*p* < 0.0001), and in NET, those older than 45 had reduced OS compared to those <40 (*p* < 0.0001) (Supplemental Table S2). A rectal primary was identified in 62% of NET cases, and 78% of AC cases presented with a colonic primary. Rectal NETs were more frequent in males than colonic NETs (50% vs. 43%, *p* < 0.0001). Colonic NETs had a higher rate of surgery than rectal NETs (92% vs. 82%, *p* < 0.0001). In the AC group, rectal AC patients were younger (mean 64 vs. 68 years, *p* < 0.0001) and more likely male (59% vs. 50%, *p* < 0.0001). Surgical resection was less frequent in rectal than colonic ACs (75% vs. 89%, *p* < 0.0001), but rectal ACs had higher rates of radiation (44% vs. 2%) than colonic ACs (*p* < 0.0001) (Supplemental Table S3).

## 4. Discussion

In this large population-based study from 2000 to 2022, differences in the clinical characteristics, treatment patterns, and outcomes for SCNEC, LCNEC, NETs, and AC were observed. Colorectal NEC is rare, comprising under 1% of colorectal cancer cases. The mean ages at diagnosis of SCNEC and LCNEC at 64 and 67 years respectively are consistent with prior work, as is the male predominance [9,17]. In particular, rectal NEC tended to be diagnosed at a younger age than colonic NEC. While demographic features such as younger age, female sex, and increased household income were associated with improved mOS for ACs and NETs, there were no demographic features that impacted OS in SCNEC or LCNEC.

Patients with colorectal NEC have a poor prognosis and in the recently reported NORDIC-NEC 2 study, the PFS and OS were 2.4 months and 6.7 months, respectively [18]. Prior work has found that colorectal SCNEC has a worse prognosis than LCNEC, with a 5-year survival of 10% for SCNEC versus 19% for LCNEC, and an associated HR of 1.3 for SCNEC [5,19]. This study cohort corroborates these findings, with a mOS of 7 months for SCNEC and 8 months for LCNEC. Advanced stage at presentation likely contributes to these worse outcomes, with 66% of the SCNEC cohort presenting with metastatic disease, compared to 55% of the LCNEC cohort. This contrasts with NET and AC, in which only 2% and 18% of cases respectively were stage IV, similar to figures obtained in prior work [16]. The later presentation of colorectal NEC may be driven by the fact that 90% of extrapulmonary NECs are non-hormone-secreting, making late-stage presentation common, as well as the aggressive nature of these cancers with increased predilection for lymphatic and vascular invasion, even in earlier stage disease [6,8,16,20].

There were several clinically meaningful survival differences between SCNEC and LCNEC based on tumor location, with colonic SCNEC emerging as the subtype with the poorest outcomes. While LCNEC was predominately colonic, at 81% colonic and 19% rectal, SCNEC was more evenly distributed at 48% colonic and 52% rectal, respectively. Rectal SCNEC patients tended to be younger and more likely to receive radiation compared to colonic SCNEC. Indeed, stage IV rectal SCNEC had a statistically significant improved mOS compared to stage IV colonic SCNEC. Although rectal LCNEC patients were similarly younger and more likely to receive radiation than colonic LCNEC patients, unlike in the SCNEC cohort, this did not result in a survival difference. In addition, when matched by stage and site of disease, stage IV colonic SCNEC patients demonstrated worse mOS compared to stage IV colonic LCNEC patients, highlighting that stage IV colonic SCNEC not only fares worse compared to stage IV rectal SCNEC, but also stage IV LCNEC at the same anatomic site. These findings are corroborated by work by Fields et al., who evaluated 1208 patients with colorectal NEC and similarly found worse survival for colonic over rectal NEC, with a HR of 1.59 [16]. Taken together, these findings suggest colonic SCNEC may be a distinct biologic entity with poorer outcomes, or suffer from reduced treatment aggressiveness, both of which warrant further investigation. As colonic NEC has been reported to harbor BRAF V600E mutations, it may be reasonable to study the value of BRAF inhibitors administered concurrently with chemotherapy [13]. Additionally, emerging molecular data further support differences between SCNEC and LCNEC, with the former having an increased prevalence of loss of RB expression and elevated p16 and Bcl-2 expression for SCNEC compared to LCNEC [21].

The role of surgical resection in colorectal NEC remains unclear, especially given the limited use of surgery in small cell lung cancer. Prior studies have demonstrated mixed outcomes regarding surgical benefit in colorectal NEC. Dasari et al. and Fields et al. found improved HR of up to 0.54 with resection in contrast to Smith et al., who saw no association between surgery and improved outcomes [16,17,22]. Shafqat et al. found improved surgical outcomes with non-small cell NEC but not SCNEC [5]. In this cohort, surgical resection was associated with improved OS in both SCNEC and LCNEC, with all tumor sites supporting a potential role for aggressive multimodal management in select patients. However, resection was only performed in 36% of SCNEC and 74% of LCNEC cases compared to 85% for both NETs and ACs. Thus, these improved outcomes could reflect fitness to undergo surgery or earlier stage disease rather than efficacy of resection itself.

Radiation therapy was uncommonly given, in 24% and 11% of the SCNEC and LCNEC cases, respectively. Its efficacy is also controversial, with prior work by Fields et al. finding no association between OS and radiation for colorectal NEC while others found durable responses following chemoradiation for localized rectal NEC [16,17,23]. In this cohort, receipt of radiation was associated with improved OS for SCNEC and LCNEC, with HRs of 0.58 and 0.71, respectively (*p* < 0.05). When stratified by site of disease, this impact was clearly driven by rectal SCNEC and LCNEC specifically. Utilizing multivariate analysis, these results only approached statistical significance with SCNEC HR = 0.79 (*p* = 0.06) and LCNEC HR = 0.59 (*p* = 0.07). Although these findings utilizing site-specific multivariate analysis did not reach conventional statistical significance, these findings regarding possible benefits of radiation in rectal NEC are hypothesis-generating and warrant further investigation.

Strengths of this study are its large sample size and extended study duration. To our knowledge, this is the largest population-based study for NEC that stratifies analysis by site of disease. However, several limitations must be acknowledged. First, the study data are limited by the retrospective nature of SEER data. Without verification of SEER pathology classification, there is potential for misclassification, such as colonic NETs that could be better classified as terminal ileum NETs. This is compounded by 2017 changes in WHO classifications incorporating Ki-67 and mitotic count for NEN classification into Grade 1, 2, or 3. Thus, patients before and after 2017 face classification changes, including between NET G3 and NEC. Additionally, mixed neuroendocrine–non-neuroendocrine neoplasms as well as appendiceal tumors may not have been reliably categorized. Due to the reported lower sensitivity and incomplete capture of chemotherapy reporting in the SEER database, as well as a lack of stratification by type of chemotherapy, this study did not evaluate the impact of chemotherapy to avoid potentially misleading conclusions due to incomplete data [24]. Future work evaluating chemotherapy efficacy in this population would be valuable. Similarly, the SEER database does not reliably differentiate surgery performed with curative intent from palliative procedures, thus introducing potential selection bias into surgical survival analyses, particularly among patients with metastatic disease. Finally, there was no detailed information about patterns of multimodal therapy or data about patients’ performance statuses, comorbidities, or biologic data such as Ki-67 or mitotic index that may better stratify groups and impact survival outcomes. Improved coding of NEC will be valuable for future similar studies. Finally, the data from SEER measures OS from all causes rather than disease-specific mortality.

## 5. Conclusions

In this large, population-based study, meaningful differences in the clinical characteristics, treatment patterns, and outcomes among patients with SCNEC, LCNEC, NETs and ACs were found. Colorectal NECs are rare but highly aggressive malignancies with markedly inferior outcomes compared to NETs and ACs. While both colorectal SCNEC and LCNEC demonstrated aggressive disease biology unaffected by demographic factors, LCNEC had a modestly better mOS than SCNEC, perhaps driven by more frequent presentation with earlier stage disease. Anatomic site played an important role in disease behavior, with rectal SCNEC and LCNEC being more common in younger patients, less likely to be metastatic, and more often managed with chemoradiation than their colonic counterparts. Additionally, stage IV colonic SCNEC demonstrated worse outcomes compared to both stage IV rectal SCNEC and stage IV colonic LCNEC. Taken together, these potential site-specific differences support the concept of SCNEC and LCNEC as biologically heterogeneous diseases and underscore the importance of these distinctions for prognostication and treatment selection. Surgery and radiation were found to be associated with improved OS in SCNEC and LCNEC. Specifically, the improved OS from radiation appears to be driven by patients with rectal disease. Although limited by the retrospective nature of the SEER database, this study represents one of the largest population-based analyses of colorectal neuroendocrine carcinomas and provides important data regarding epidemiologic and survival data for these rare malignancies. Further research should investigate the molecular differences between rectal and colonic NEC, tools to improve early detection, and the utility of combined treatment modalities and inclusion of molecularly directed therapy for these rare, aggressive malignancies.

## Figures and Tables

**Table 1:** NEC Demographics.

Variable	SCNEC (*N* = 790)	LCNEC (*N* = 498)
Age at diagnosis (continuous)	64 (14); 65, 24, 89	67 (13); 68, 19, 89
Sex		
Female	353 (45%)	243 (49%)
Male	437 (55%)	255 (51%)
Race		
White	660 (84%)	412 (83%)
Black	87 (11%)	49 (10%)
Other	43 (5%)	35 (7%)
Anatomic Site		
Appendix	9 (1%)	14 (3%)
Cecum	121 (15%)	120 (24%)
Ascending Colon	103 (13%)	103 (21%)
Hepatic Flexure	25 (3%)	24 (5%)
Transverse Colon	33 (4%)	53 (11%)
Splenic Flexure	10 (1%)	9 (2%)
Descending Colon	12 (2%)	12 (2%)
Sigmoid Colon	42 (5%)	45 (9%)
Rectosigmoid Junction	26 (3%)	25 (5%)
Rectum	409 (52%)	93 (19%)
AJCC stage *		
Stage I	46 (6%)	27 (5%)
Stage II	34 (4%)	33 (7%)
Stage III	92 (12%)	144 (29%)
Stage IV	520 (66%)	274 (55%)
Unknown	98 (12%)	20 (4%)
T stage *		
T0	1 (0%)	0 (0%)
T1	101 (13%)	44 (9%)
T2	28 (4%)	30 (6%)
T3	167 (21%)	193 (39%)
T4	170 (22%)	150 (30%)
Tis	3 (0%)	0 (0%)
Unknown	320 (41%)	81 (16%)
N stage *		
N0	208 (26%)	112 (22%)
N1	154 (19%)	139 (28%)
N2	133 (17%)	178 (36%)
N3	9 (1%)	2 (0%)
Unknown	286 (36%)	67 (13%)
M stage *		
M0	242 (31%)	221 (44%)
M1	520 (66%)	274 (55%)
Unknown	28 (4%)	3 (1%)
Surgery		
No Surgery	503 (64%)	130 (26%)
Local Excision	34 (4%)	17 (3%)
Partial Resection	77 (10%)	96 (20%)
Subtotal/Extended Resection	139 (18%)	231 (47%)
Total Resection	30 (4%)	15 (3%)
Other/Unknown Surgery	6 (1%)	2 (0%)
Radiation		
Yes	188 (24%)	53 (11%)
No/Unknown	602 (76%)	445 (89%)

* At diagnosis.

**Table 2:** NEC Site-Specific Demographics.

	SCNEC			LCNEC		
Variable	Colon *N* = 355	Rectum *N* = 435	*p*-Value	Colon *N* = 380	Rectum *N* = 118	*p*-Value
Age at diagnosis (continuous) ^1^	66 (13) 67, 24, 89	62 (14) 63, 24, 89	0.0004	67 (13) 69, 19, 89	64 (14) 66, 25, 88	0.0289
Sex ^2^			0.7875			0.0336
Male	194 (55%)	243 (56%)		184 (48%)	71 (60%)	
Female	161 (45%)	192 (44%)		196 (52%)	47 (40%)	
Race ^2^			0.3165			0.0501
White	294 (83%)	366 (84%)		317 (84%)	95 (81%)	
Black	37 (10%)	50 (11%)		40 (11%)	9 (7.6%)	
Other	24 (6.8%)	19 (4.4%)		21 (5.6%)	14 (12%)	
AJCC stage *^2^			0.6533			0.0461
Stage I	19 (5.7%)	27 (7.5%)		17 (4.6%)	10 (9.3%)	
Stage II	17 (5.1%)	17 (4.7%)		30 (8.1%)	3 (2.8%)	
Stage III	41 (12%)	51 (14%)		107 (29%)	37 (35%)	
Stage IV	256 (77%)	264 (74%)		217 (58%)	57 (53%)	
T stage *^3^			0.0605			0.0005
T2	16 (4.5%)	12 (2.8%)		18 (4.7%)	12 (10%)	
T3	73 (21%)	94 (22%)		154 (41%)	39 (33%)	
T4	89 (25%)	81 (19%)		132 (35%)	18 (15%)	
Tis/T0/T1	38 (11%)	67 (15%)		28 (7.4%)	16 (14%)	
Unknown	139 (39%)	181 (42%)		48 (13%)	33 (28%)	
N stage *^3^			0.0005			0.0005
N0	90 (25%)	118 (27%)		78 (21%)	34 (29%)	
N1	60 (17%)	94 (22%)		105 (28%)	34 (29%)	
N2	98 (28%)	35 (8.0%)		156 (41%)	22 (19%)	
N3	6 (1.7%)	3 (0.7%)		2 (0.5%)	0 (0%)	
Unknown	101 (28%)	185 (43%)		39 (10%)	28 (24%)	
M stage *^2^			0.0034			0.2369
M0	89 (25%)	153 (35%)		161 (42%)	60 (51%)	
M1	256 (72%)	264 (61%)		217 (57%)	57 (48%)	
Unknown	10 (2.8%)	18 (4.1%)		2 (0.5%)	1 (0.8%)	
Surgery ^2^			<0.0001			<0.0001
Yes	203 (57%)	84 (19%)		312 (82%)	56 (47%)	
No	152 (43%)	351 (81%)		68 (18%)	62 (53%)	
Radiation ^2^			<0.0001			<0.0001
Yes	22 (6.2%)	166 (38%)		13 (3.4%)	40 (34%)	
No/Unknown	333 (94%)	269 (62%)		367 (97%)	78 (66%)	

Notes: ^1^ *t*-test; age presented as mean (SD), median, minimum, maximum. ^2^ Chi-square test (unknowns excluded from test). ³ Fisher’s exact test with simulate.p.value = TRUE (unknowns excluded from test). Unknowns were displayed but excluded from statistical tests. T0 and Tis were combined into a single category. Rectum category includes both rectum and rectosigmoid junction as defined in the Site recode ICD-O-3/WHO 2008 variable in SEER. * At diagnosis.

**Table 3:** NEC Survival Outcomes Based on Histology and Site of Disease.

Median Overall Survival (Months)	Stage I	Stage II	Stage III	Stage IV
Colon SCNEC (n/mOS)	19/32	17/38	40/14	245/2
Colon LCNEC (n/mOS)	17/122	30/49	107/13	217/5
SCNEC vs. LCNEC in Colon: *p*-value	0.25	0.65	0.48	<0.0001
Rectal SCNEC (n/mOS)	27/23	17/16	50/19	260/7
Rectal LCNEC (n/mOS)	10/28	3/NA	37/17	57/6
SCNEC vs. LCNEC in Rectum: *p*-value	0.88	0.3	0.45	0.25
Colon SCNEC vs. Rectal SCNEC *p*-value	0.99	0.065	0.48	<0.0001
Colon LCNEC vs. Rectal LCNEC: *p*-value	0.24	0.55	0.47	0.95

**Table 4:** Multivariate Analysis of Variables Impacting Survival by Histology and Site of Disease.

**SCNEC**							
		Colon			Rectum		
Variable	Term	HR	95% CI	*p*-Value	HR	95% CI	*p*-Value
Multivariate (AJCC)							
AJCC Stage *	Stage I (reference)						
	Stage II	0.72	0.28–1.89	0.5067	1.78	0.82–3.86	0.146
	Stage III	3.25	1.62–6.51	0.0009	1.85	1–3.45	0.051
	Stage IV	11.79	6.09–22.83	<0.0001	4.53	2.54–8.1	<0.0001
Surgery	Yes (reference)						
	No	1.8	1.39–2.33	<0.0001	1.91	1.36–2.7	0.0002
Radiation	Yes (reference)						
	No/Unknown				1.27	0.99–1.63	0.0565
Multivariate (TNM)							
T Stage *	T1/T2 (reference)						
	T3	0.74	0.46–1.19	0.2186	1.41	0.96–2.08	0.0808
	T4	1.03	0.64–1.64	0.9165	2.33	1.55–3.5	<0.0001
M Stage *	M0 (reference)						
	M1	3.56	2.24–5.64	<0.0001	2.04	1.47–2.82	<0.0001
N Stage *	N0 (reference)						
	N1	2.86	1.7–4.81	<0.0001			
	N2	3.33	1.95–5.68	<0.0001			
	N3	4.76	0.6–37.66	0.1398			
Surgery	Yes (reference)						
	No	2.81	1.69–4.67	<0.0001	2.09	1.43–3.06	0.0001
Radiation	Yes (reference)						
	No/Unknown				1.29	0.94–1.78	0.1137
**LCNEC**							
		Colon			Rectum		
Variable	Term	HR	95% CI	*p*-value	HR	95% CI	*p*-value
Multivariate (AJCC)							
AJCC Stage *	Stage I (reference)						
	Stage II	1.94	0.76–4.94	0.163	0.96	0.11–8.53	0.9731
	Stage III	4.17	1.8–9.65	0.0009	5.05	1.97–12.94	0.0007
	Stage IV	10.99	4.76–25.41	<0.0001	7.76	2.84–21.15	<0.0001
Surgery	Yes (reference)						
	No	2.07	1.5–2.85	<0.0001	3.79	1.87–7.69	0.0002
Radiation	Yes (reference)						
	No/Unknown				1.75	0.94–3.26	0.0783
T Stage *	T1/T2 (reference)						
Multivariate (TNM)							
	T3	1.31	0.81–2.13	0.2728	3.59	1.67–7.72	0.0011
	T4	2.08	1.28–3.38	0.0031	6.73	2.61–17.36	<0.0001
M Stage *	M0 (reference)						
	M1	2.72	2.01–3.69	<0.0001	2.38	1.09–5.21	0.0296
N Stage *	N0 (reference)						
	N1	1.58	1.04–2.41	0.0337			
	N2	2.35	1.54–3.6	<0.0001			
	N3	2.24	0.3–16.67	0.4292			
	Unknown						
Surgery	Yes (reference)						
	No	4.04	2.11–7.74	<0.0001	2.55	1.22–5.29	0.0123
Radiation	Yes (reference)						
	No/Unknown				1.39	0.59–3.27	0.4465

* At diagnosis.

## Data Availability

The original data presented in the study are openly available at https://seer.cancer.gov/data/ (accessed on the 1 August 2025).

## Supplementary Materials

The following supporting information can be downloaded at: https://www.mdpi.com/article/10.3390/cancers18121976/s1, Table S1: NET and AC Demographics; Table S2: NET and AC Demographics By Location; Table S3: NET and AC Survival Outcomes Based on Histology and Site of Disease; Table S4: Multivariate Analysis of Variables Impacting Survival by Histology and Site of Disease.
