# Supplementary material for: Small Cell and Large Cell Neuroendocrine Carcinoma of the Colon and Rectum: Population-Based Analysis of Incidence, Survival, and Site-Specific Outcomes
Source: Cancers (Basel). 2026 Jun 18;18(12):1976. doi: 10.3390/cancers18121976 (PMC13297598; doi:10.3390/cancers18121976)
Supplement: Supplementary file 1 [file cancers-18-01976-s001.zip › cancers-4318414-supplementary.pdf]

Supplemental Tables

Supplemental Table S1. NET and AC Demographics.

Variable	NET (N = 31,200)	Adenocarcinoma (N = 677287)
Age at diagnosis (continuous)	53 (15); 53, 0, 89	66 (14); 67, 0, 89
Sex		
Female	16,412 (53%)	320,748 (47%)
Male	14,788 (47%)	356,539 (53%)
Race		
White	19,836 (67%)	532,308 (79%)
Black	5,914 (20%)	75,599 (11%)
Other	3,951 (13%)	63,779 (9%)
Anatomic Site		
Appendix	7,071 (23%)	14,588 (2%)
Cecum	1,741 (6%)	101,996 (15%)
Ascending Colon	328 (1%)	89,048 (13%)
Hepatic Flexure	46 (0%)	23,044 (3%)
Transverse Colon	71 (0%)	44,066 (7%)
Splenic Flexure	22 (0%)	15,469 (2%)

Descending Colon	114 (0%)	29,459 (4%)
Sigmoid Colon	1,442 (5%)	143,733 (21%)
Rectosigmoid Junction	968 (3%)	53,885 (8%)
Rectum	19,397 (62%)	161,999 (24%)
AJCC stage*		
Stage I	9,683 (31%)	160,722 (24%)
Stage II	292 (1%)	155,399 (23%)
Stage III	685 (2%)	146,985 (22%)
Stage IV	553 (2%)	116,734 (17%)
Unknown	19,987 (64%)	97,447 (14%)
T stage*		
T0	5 (0%)	218 (0%)
T1	10,825 (35%)	120,594 (18%)
T2	809 (3%)	80,754 (12%)
T3	609 (2%)	277,729 (42%)
T4	353 (1%)	82,093 (12%)
Tis	214 (1%)	20,968 (3%)
Unknown	18,385 (59%)	78,135 (12%)

N stage*		
N0	12,323 (39%)	382,870 (58%)
N1	884 (3%)	129,799 (20%)
N2	25 (0%)	68,269 (10%)
N3	41 (0%)	4,409 (1%)
Unknown	17,927 (57%)	75,144 (11%)
M stage*		
M0	16,043 (51%)	529,205 (80%)
M1	553 (2%)	116,734 (18%)
Unknown	14,604 (47%)	14,552 (2%)
Surgery		
No Surgery	4,528 (15%)	103,253 (15%)
Local Excision	17,150 (55%)	62,288 (9%)
Partial Resection	6,010 (19%)	246,004 (37%)
Subtotal/Extended Resection	2,597 (8%)	225,039 (34%)
Total Resection	213 (1%)	28,311 (4%)
Other/Unknown Surgery	616 (2%)	6,573 (1%)
Radiation		

Yes	152 (0%)	94,621 (14%)
No/Unknown	31,048 (100%)	582,666 (86%)

* At diagnosis

Supplemental Table S2. NET and AC Demographics By Location.

	NETs			Adenocarcinoma		
**Variable**	**Colon N = 10,835**	**Rectum N = 20,365**	**p-value**	**Colon N = 461,403**	**Rectum N = 215,884**	**p-value**
Age at diagnosis (continuous)[1]	48 (19)<br>50, 0, 89	55 (11)<br>54, 12, 89	<0.0001	67 (14)<br>68, 0, 89	63 (13)<br>63, 6, 89	<0.0001
Sex[2]			<0.0001			<0.0001
Male	4,631 (43%)	10,157 (50%)		230,998 (50%)	125,541 (58%)	
Female	6,204 (57%)	10,208 (50%)		230,405 (50%)	90,343 (42%)	
Race[2]			<0.0001			<0.0001
White	8,789 (83%)	11,047 (58%)		364,323 (79%)	167,985 (79%)	
Black	1,179 (11%)	4,735 (25%)		54,128 (12%)	21,471 (10%)	
Other	575 (5.5%)	3,376 (18%)		39,972 (8.7%)	23,807 (11%)	
AJCC stage[2]*			<0.0001			<0.0001
Stage I	4,497 (78%)	5,186 (95%)		108,120 (26%)	52,602 (31%)	

Stage II	259 (4.5%)	33 (0.6%)		118,221 (29%)	37,178 (22%)	
Stage III	582 (10%)	103 (1.9%)		101,496 (25%)	45,489 (27%)	
Stage IV	395 (6.9%)	158 (2.9%)		81,208 (20%)	35,526 (21%)	
T stage[3]*			0.0005			<0.0001
T2	470 (4.3%)	339 (1.7%)		53,998 (12%)	26,756 (13%)	
T3	507 (4.7%)	102 (0.5%)		197,457 (43%)	80,272 (39%)	
T4	331 (3.1%)	22 (0.1%)		63,281 (14%)	18,812 (9.2%)	
Tis/T0/T1	5,077 (47%)	5,967 (29%)		92,632 (20%)	49,148 (24%)	
Unknown	4,450 (41%)	13,935 (68%)		48,365 (11%)	29,770 (15%)	
N stage[3]*			0.0005			<0.0001
N0	5,215 (48%)	7,108 (35%)		269,266 (59%)	113,604 (55%)	
N1	708 (6.5%)	176 (0.9%)		88,114 (19%)	41,685 (20%)	
N2	21 (0.2%)	4 (<0.1%)		51,178 (11%)	17,091 (8.3%)	
N3	40 (0.4%)	1 (<0.1%)		3,841 (0.8%)	568 (0.3%)	
Unknown	4,851 (45%)	13,076 (64%)		43,334 (9.5%)	31,810 (16%)	
M stage[2]*			<0.0001			<0.0001

M0	6,803 (63%)	9,240 (45%)		365,813 (80%)	163,392 (80%)	
M1	395 (3.6%)	158 (0.8%)		81,208 (18%)	35,526 (17%)	
Unknown	3,637 (34%)	10,967 (54%)		8,712 (1.9%)	5,840 (2.9%)	
Surgery[2]*			<0.0001			<0.0001
Yes	10,014 (92%)	16,658 (82%)		410,143 (89%)	163,891 (76%)	
No	821 (7.6%)	3,707 (18%)		51,260 (11%)	51,993 (24%)	
Radiation[2]*			0.0032			<0.0001
Yes	35 (0.3%)	117 (0.6%)		9,746 (2.1%)	84,875 (39%)	
No/Unknown	10,800 (100%)	20,248 (99%)		451,657 (98%)	131,009 (61%)	

Notes:
[1] t-test; age presented as mean (SD), median, minimum, maximum
[2] Chi-square test (unknowns excluded from test)
* At diagnosis
• Unknowns were displayed but excluded from statistical tests.
• T0 and Tis were combined into a single category.
• Rectum category includes both rectum and rectosigmoid junction as defined in the Site recode ICD-O-3/WHO 2008 variable in SEER.

Supplemental Table S3. NET and AC Survival Outcomes Based on Histology and Site of Disease.

**Median overall survival (months)**	**Stage I**	**Stage II**	**Stage III**	**Stage IV**
Colon NETs (n/mOS)	4496/NA	259/NA	582/184	393/49
Colon AC (n/mOS)	114,213/144	121,757/106	104,793/82	83,542/13
Rectal NETs (n/mOS)	5185/NA	33/NA	103/102	158/36

Rectal AC (n/mOS)	54,702/160	38,417/106	46,687/107	36,652/17

Supplemental Table S4. Multivariate Analysis of Variables Impacting Survival by Histology and Site of Disease.

NETs

		**Colon**			**Rectum**		
**Variable**	**Term**	**HR**	**95% CI**	**P-value**	**HR**	**95% CI**	**P-value**
Multivariate (AJCC)							
Age Group	<40 (reference)						
	40-44	6.42	3.11–13.25	<0.0001	1.54	0.79–3.04	0.2077
	45-49	8.95	4.65–17.22	<0.0001	2.13	1.19–3.82	0.0107
	50-54	8.96	4.83–16.62	<0.0001	2.25	1.33–3.81	0.0025
	55-59	12.59	6.89–22.99	<0.0001	3.22	1.91–5.45	<0.0001
	60-64	17	9.37–30.81	<0.0001	4.98	2.96–8.38	<0.0001
	65-69	30.71	17.21–54.79	<0.0001	7.96	4.77–13.28	<0.0001
	70+	51.55	29.46–90.2	<0.0001	15.45	9.37–25.49	<0.0001
Sex	Male (reference)						
	Female	0.85	0.72–0.99	0.0391	0.79	0.68–0.93	0.0044
Race	White (reference)						
	Black	1.24	0.97–1.58	0.084	1.25	1.04–1.51	0.0192
	Other (American Indian/AK Native,	1.41	0.98–2.04	0.0659	0.91	0.72–1.15	0.4407

	Asian/Pacific Islander)						
AJCC Stage*	Stage I (reference)						
	Stage II	1.4	1.01–1.92	0.0408	1.96	0.95–4.02	0.0676
	Stage III	1.18	0.93–1.48	0.1682	2.8	1.73–4.53	<0.0001
	Stage IV	3.07	2.49–3.78	<0.0001	9.03	6.53–12.49	<0.0001
Surgery	Yes (reference)						
	No	2.15	1.65–2.79	<0.0001	1.45	1.12–1.86	0.0043
Radiation	Yes (reference)						
	No/Unknown	0.75	0.37–1.55	0.4425	0.92	0.54–1.56	0.754
Multivariate (TMN)							
Age Group	<40 (reference)						
	40-44	5.04	2.51–10.1	<0.0001	1.55	0.83–2.88	0.1713
	45-49	6.38	3.38–12.04	<0.0001	1.9	1.09–3.31	0.0233
	50-54	7.51	4.28–13.19	<0.0001	2.17	1.34–3.53	0.0017
	55-59	11.56	6.69–19.95	<0.0001	2.97	1.82–4.84	<0.0001
	60-64	17.3	10.17–29.41	<0.0001	5.28	3.27–8.55	<0.0001
	65-69	28	16.66–47.05	<0.0001	8.26	5.15–13.25	<0.0001
	70+	51.27	31.33–83.89	<0.0001	16.08	10.13–25.52	<0.0001
Sex	Male (reference)						
	Female	0.86	0.73–1.01	0.0592	0.78	0.67–0.91	0.0014
Race	White (reference)						
	Black	1.16	0.9–1.49	0.2596	1.22	1.01–1.47	0.0352

	Other (American Indian/AK Native, Asian/Pacific Islander)	1.32	0.92–1.88	0.1337	0.89	0.72–1.11	0.3009
T Stage*	T1/T2 (reference)						
	T3	1.24	0.98–1.56	0.0689	1.72	1.05–2.82	0.031
	T4	1.91	1.44–2.52	<0.0001	1.67	0.76–3.68	0.1995
M Stage*	M0 (reference)						
	M1	1.91	1.34–2.72	0.0003	1.14	0.65–2	0.6385
N Stage*	N0 (reference)				12.2	1.55–95.85	0.0174
	N1	0.87	0.67–1.13	0.3045	15.91	2.03–124.6	0.0084
	N2	1.11	0.6–2.08	0.7379	0.88	0.72–1.07	0.1999
	N3	1.28	0.75–2.18	0.3584			
	Unknown	1.2	0.97–1.49	0.0965	5.11	2.75–9.47	<0.0001
Surgery	Yes (reference)						
	No	1.84	1.21–2.8	0.0045	1.35	1.04–1.75	0.0246
Radiation	Yes (reference)						
	No/Unknown	0.54	0.2–1.48	0.2321	0.71	0.35–1.43	0.336

* At diagnosis

Adenocarcinoma

		Colon			Rectum		
**Variable**	**Term**	**HR**	**95% CI**	**P-value**	**HR**	**95% CI**	**P-value**
Multivariate (AJCC)							
Age Group	<40 (reference)						

	40-44	0.99	0.93–1.05	0.7224	0.96	0.91–1.01	0.0877
	45-49	1.03	0.98–1.09	0.2177	1.09	1.05–1.14	<0.0001
	50-54	1.06	1–1.11	0.0329	1.05	1.01–1.1	0.0093
	55-59	1.24	1.18–1.31	<0.0001	1.27	1.22–1.32	<0.0001
	60-64	1.48	1.41–1.56	<0.0001	1.49	1.43–1.54	<0.0001
	65-69	1.78	1.69–1.86	<0.0001	1.79	1.72–1.86	<0.0001
	70+	3.17	3.03–3.31	<0.0001	3.27	3.15–3.39	<0.0001
Sex	Male (reference)						
	Female	1.15	1.14–1.17	<0.0001	1.13	1.12–1.13	<0.0001
Race	White (reference)						
	Black	1.22	1.19–1.24	<0.0001	1.13	1.11–1.14	<0.0001
	Other (American Indian/AK Native, Asian/Pacific Islander)	0.92	0.9–0.95	<0.0001	0.85	0.83–0.86	<0.0001
AJCC Stage*	Stage I (reference)						
	Stage II	1.67	1.63–1.71	<0.0001	1.41	1.4–1.43	<0.0001
	Stage III	2.13	2.08–2.17	<0.0001	2.4	2.37–2.43	<0.0001
	Stage IV	7.05	6.88–7.22	<0.0001	9.6	9.45–9.75	<0.0001
Surgery	Yes (reference)						
	No	2.79	2.74–2.85	<0.0001	2.55	2.51–2.59	<0.0001
Radiation	Yes (reference)						
	No/Unknown	0.97	0.96–0.99	0.0012	0.77	0.75–0.79	<0.0001
Multivariate (TMN)							

Age Group	<40 (reference)						
	40-44	0.96	0.9–1.03	0.2868	0.96	0.91–1.01	0.1092
	45-49	1.06	1–1.12	0.0612	1.11	1.06–1.16	<0.0001
	50-54	1.08	1.03–1.14	0.004	1.09	1.05–1.14	<0.0001
	55-59	1.32	1.25–1.39	<0.0001	1.36	1.3–1.42	<0.0001
	60-64	1.62	1.53–1.7	<0.0001	1.62	1.55–1.69	<0.0001
	65-69	1.96	1.86–2.06	<0.0001	2	1.92–2.09	<0.0001
	70+	3.71	3.54–3.9	<0.0001	3.85	3.7–4.01	<0.0001
Sex	Male (reference)						
	Female	1.17	1.15–1.19	<0.0001	1.15	1.14–1.16	<0.0001
Race	White (reference)						
	Black	1.21	1.18–1.24	<0.0001	1.15	1.13–1.16	<0.0001
	Other (American Indian/AK Native, Asian/Pacific Islander)	0.89	0.87–0.91	<0.0001	0.83	0.82–0.85	<0.0001
T Stage*	T1/T2 (reference)						
	T3	1.43	1.41–1.46	<0.0001	1.36	1.34–1.37	<0.0001
	T4	2.22	2.16–2.27	<0.0001	2.08	2.05–2.11	<0.0001
M Stage*	M0 (reference)						
	M1	1.21	1.19–1.23	<0.0001	1.5	1.48–1.52	<0.0001
N Stage*	N0 (reference)	1.73	1.68–1.77	<0.0001	2.18	2.15–2.22	<0.0001
	N1	1.58	1.42–1.75	<0.0001	1.79	1.72–1.87	<0.0001
	N2	1.29	1.26–1.33	<0.0001	1.55	1.52–1.58	<0.0001

	N3						
	Unknown	3.08	3.01–3.15	<0.0001	3.78	3.72–3.83	<0.0001
Surgery	Yes (reference)						
	No	2.96	2.9–3.03	<0.0001	3.61	3.53–3.69	<0.0001
Radiation	Yes (reference)						
	No/Unknown	0.98	0.96–1	0.0342	0.8	0.77–0.82	<0.0001

* At diagnosis
